# Olive Oil Phenolics Prevent Oxysterol‐Induced Proinflammatory Cytokine Secretion and Reactive Oxygen Species Production in Human Peripheral Blood Mononuclear Cells, Through Modulation of p38 and JNK Pathways

**DOI:** 10.1002/mnfr.201700283

**Published:** 2017-10-26

**Authors:** Gessica Serra, Monica Deiana, Jeremy P. E. Spencer, Giulia Corona

**Affiliations:** ^1^ Department of Food and Nutritional Sciences University of Reading Reading UK; ^2^ Department of Biomedical Sciences University of Cagliari Cagliari Italy; ^3^ Health Sciences Research Centre University of Roehampton London UK

**Keywords:** inflammation, MAPK, olive oil polyphenols, oxysterols, PBMCs

## Abstract

**Scope:**

The aim of the present study was to investigate the ability of extra virgin olive oil (EVOO) polyphenols to counteract the proinflammatory effects induced by dietary and endogenous oxysterols in ex vivo immune cells.

**Methods and results:**

Peripheral blood mononuclear cells (PBMCs), separated from the whole blood of healthy donors, were utilized and were stimulated with an oxysterols mixture, in the presence of physiologically relevant concentrations of the EVOO polyphenols, hydroxytyrosol, tyrosol, and homovanillic alcohol. Oxysterols significantly increased the production of proinflammatory cytokines, interleukin‐1β, regulated on activation, normal T‐cell expressed and secreted and macrophage migration inhibitory factor in ex vivo cultured PBMCs. Increased levels of reactive oxygen species (ROS) were also detected along with increased phosphorylation of the p38 and JNK. All phenolic compounds significantly reduced cytokine secretion induced by the oxysterols and inhibited ROS production and mitogen activated protein kinase phosphorylation.

**Conclusions:**

These results suggest that extra virgin olive oil polyphenols modulate the immune response induced by dietary and endogenous cholesterol oxidation products in human immune cells and may hold benefit in controlling chronic immune and/or inflammatory processes.

## 1 Introduction

Oxysterols are 27‐carbon‐atom molecules resulting from nonenzymatic or enzymatic oxidation of cholesterol and have been detected in plasma and tissues due to endogenous formation and dietary intake.^1^, ^2^, ^3^ High levels of oxysterols, generated by dysfunction in endogenous production or through high dietary intake, can affect cellular metabolism, change membrane composition/property, and promote the onset and progression of major chronic and degenerative diseases such as cancer and atherosclerosis.^4^, ^5^ It is thought that such deleterious effects are due to their ability to trigger cytotoxic, prooxidative, and proinflammatory reactions,^6^ such as the production of superoxide anions (O^−2^) and reactive oxygen species (ROS), or through their potential to enhance proinflammatory cytokine expression and secretion levels (tumor necrosis factor‐α (TNF‐α), IL‐1β, IL‐6, IL‐8, monocyte inflammatory protein‐1 (MIP‐1β), cellular adhesion molecule‐1 (ICAM‐1), vascular cell adhesion molecule‐1 (VCAM‐1) and E‐selectin.^7^ Notably, the expression of these and other inflammatory mediators is closely dependent on the activity nuclear factor kappa‐light‐chain‐enhancer of activated B cells (NF‐κb), through activation of the mitogen‐activated protein kinase (MAPK) pathways.^8^

Extra virgin olive oil, the main fat source in the Mediterranean diet, with its high content in monounsaturated fatty acids and relatively high polyphenol content, may attenuate such inflammatory responses and exert beneficial effects in modulating chronic low‐grade inflammation.^9^, ^10^, ^11^, ^12^, ^13^ Extra virgin olive oil (EVOO) and its bioactive minor components, in particular, oleuropein,^14^, ^15^ hydroxytyrosol,^16^, ^17^, ^18^, ^19^, ^20^, ^21^ and oleocanthal,^22^, ^23^ but also the entire phenolic fraction,^24^, ^25^ have been largely investigated in in vitro models and many findings support their anti‐inflammatory and immune‐modulatory effects.

This is thought to occur through their capacity to limit the ROS and nitrogen species formation^16^, ^24^, ^25^, ^26^, ^27^, ^28^ and to inhibit the proinflammatory activity of ROS‐generating enzymes including cyclooxygenase‐2 (COX‐2),^18^, ^24^, ^25^, ^27^, ^28^ inducible nitric oxide synthase (iNOS)^18^, ^20^, ^24^, ^25^, ^28^, ^29^ and to modulate different intracellular signaling pathways from nuclear factor kappa‐light‐chain‐enhancer of activated B cells (NF‐κB) to MAPK through perturbation of redox‐sensible networks in immune cells.^26^

Also many in vivo studies have suggested that EVOO with high phenolic concentration is effective in modulating inflammatory mediator derived from arachidonic acid, such as TBX_2_ and LTB_4_ 30, 31, 32, 33 as well as other inflammatory markers, such as high‐sensitivity C‐reactive protein,^13^, ^34^ IL‐6,^10^, ^34^, ^35^ IL‐β,^36^ IFN‐λ, and IL‐7,^30^, ^37^ VCAM‐1, ICAM‐1, TNF‐α, and monocyte chemoattractant protein‐1.^10^

The aim of this study was to investigate the ability of different olive oil pure phenolic compounds, (HT, TYR (tyrosol), and HVA), found in the blood after ingestion and absorption of EVOO, to prevent inflammatory effects induced by oxysterols in human immune cells, and to understand the implicated mechanism of action.

Peripheral blood mononuclear cells (PBMCs), separated from whole blood of healthy donors, were used for this purpose; these cells are a good model to study the inflammatory responses ex vivo since they are composed by lymphocytes, monocytes, and macrophages, critical components in the immune system to fight inflammation and source of proinflammatory molecules.

PBMCs were treated with an oxysterols mixture composed by the most widely represented oxysterols in plasma of hypercholesterolemic subjects: 7α‐hydroxycholesterol (7α‐HC), 7β‐hydroxycholesterol (7β‐HC), 7‐ketocholesterol (7‐KC), cholesterol 5α,6α‐epoxide (5α,6α‐EC), cholesterol 5β,6β‐epoxide (5β,6β‐EC), cholestane‐3β,5α,6β‐triol (triol), and 25‐hydroxycholesterol (25‐HC)^38^ at pathologically relevant concentration (20 μM). PBMCs were used in order to investigate the ability of the olive oil simple phenols to inhibit the increase of ROS and proinflammatory cytokine/chemokine synthesis (IL‐1β, MIF (macrophage migration inhibitory factor), and RANTES (regulated on activation, normal T‐cell expressed and secreted)) induced by the oxysterols mixture, and to modulate the signaling pathways (MAPK) involved in these processes.

## 2 Experimental Section

### 2.1 Reagents

Media and supplements were purchased from Lonza (Slough, UK). Histopaque‐1077, oxysterols, 2′,7′‐dichlorofluorescein diacetate (DCFH‐DA), horseradish peroxidase‐conjugated goat anti‐rabbit secondary antibody, Bradford reagent, and solvents were purchased from Sigma Aldrich (Poole, UK). The cytokine kits were purchased from R&D systems (Abingdon, UK). Gels and all material for electrophoresis and immunoblotting were obtained from Invitrogen (Milan, Italy). The Western Blotting System was from Bio‐Rad (Milan, Italy). The primary antibodies were purchased from Millipore (Watford, UK).

### 2.2 Isolation and Culture of Human Peripheral Blood Mononuclear Cells

The study was approved by the Ethics and Research Committee of the University of Reading (Project No. 12/16) and informed consent obtained from each blood donor.

The volunteers (males and females, aged between 23 and 40 years) were enrolled according to specific inclusion (signed informed consent, men, and women aged 20–40 years, nonsmokers, good general health) and exclusion criteria (history of drug abuse, including alcohol, participation in experimental trials within 3 months prior to study, use of antibiotics within the previous 3 months, use of prescribed medication, regular use of anti‐inflammatory drugs, any kind of inflammatory, auto‐immune disease or allergy, any other pathology).

Overnight fasting (12 hours) venous blood samples from 14 healthy donors were collected in the morning in sodium‐heparin coated tubes (Greiner Bio‐One Limited, Gloucestershire, UK).

PBMCs were immediately isolated by a Ficoll‐Hypaque (Histopaque‐1077) density gradient from 5 ml of whole peripheral blood following the manufacturer instructions and re‐suspended in the culture medium consisting of RPMI 1640, 1% v/v glutamine and 1% v/v antibiotics, supplemented with autologous plasma (2.5% v/v). PBMCs were counted and cultured at 37°C in a 5% CO_2_ humidified atmosphere.

### 2.3 Cell Treatments for Cytokines Analysis

PBMCs (1 × 10^6^ cells/mL) were seeded in 24‐well plate in complete RPMI, pretreated or not with HT, TYR, and homovanillic alcohol (HVA) (0.25, 0.5, 1 μM) and incubated for 30 min at 37°C in a 5% CO_2_ atmosphere. Then, the oxysterols mixture, 20 μM in ethanol, was added in the medium for 24 h.

An equivalent amount of ethanol was added to the control cells for all the treatments.

At the end of the incubation period well contents were removed, transferred to eppendorf tubes and centrifuged to pellet the cells at 400 × g for 5 min at 20°C. The supernatants were collected in clean tubes and stored at –20°C until analysis. The percentage composition of the oxysterols mixture used was: 7α‐HC (5%), 7β‐HC (10%), 5α,6α‐EC (20%), 5β,6β‐EC (20%), triol (9%), 7‐KC (35%), and 25‐HC (1%).

### 2.4 Measurement of Cytokine/Chemokines Production

Cytokines were preliminarily measured in cell culture supernatants (400 μL) of two samples, control and treated with the oxysterols mixture, 20 μM in ethanol, by a semi‐quantitative method to simultaneously detect the relative levels of 36 different cytokines and chemokines using the *Human Cytokine Array Panel A* (R&D systems, Abingdon, UK) following the manufacturer instructions. The membranes were developed using ImageQuant LAS 4000 mini. Signal intensities of each membrane array were analyzed using the ImageQuant software (Molecular Dynamics, Amersham Pharmacia Biotech). Three cytokines were selected for quantitative analysis by ELISA on the basis of the initial screening process described earlier. Levels of human cytokines IL‐1β, MIF, and RANTES were quantified using appropriate kits (*“Human IL‐1β/IL‐1F2 DuoSet*,” “*Human MIF DuoSet*,” and “*Human CCL5/RANTES DuoSet”* R&D systems, Abingdon, UK), following the manufacturer's instructions.

### 2.5 Measurement of Intracellular ROS Production

Intracellular ROS production was measured using the probe DCFH‐DA.

PBMCs (1 × 10^6^ cells/mL) were seeded in 24‐well plate in complete RPMI. In the first set of experiments cells were incubated for 30 min with DCFH‐DA 10 μM in the dark at 37°C. DCFH‐DA was then removed, and cells were washed with PBS and incubated with the oxysterols mixture, 20 μM in ethanol, added in fresh medium. DCFH‐DA loaded cells were immediately placed in a plate reader (Plate reader, Infinite 200, GENios TECAN) setting the excitation filter at 485 nm and the emission filter at 530 nm, with temperature maintained at 37°C. ROS production was monitored by reading the fluorescence emitted taking readings at intervals of 30 min for 3 h. In the second set of experiments cells were pre‐treated or not with pure phenolic compounds, HT, TYR, and HVA (0.25, 0.5, 1 μM) and incubated for 30 min at 37°C in a 5% CO_2_ atmosphere. Cells were then washed, treated with DCFH‐DA 10 μM and incubated for 30 min in the dark at 37°C. DCFH‐DA was then removed; cells were washed and incubated with the oxysterols mixture 20 μM added in fresh medium for 2 h. The fluorescence emitted from the cells was measured with the same method for 2 h.

### 2.6 Isolation of Proteins and Western Blot Analysis of JNK 1/2 and p38

PBMCs (5 × 10^6^ cells/mL) were seeded in 6‐well plate in complete RPMI, pretreated or not with HT, TYR, and HVA (0.25, 0.5, 1 μM) and incubated for 30 min at 37°C in a 5% CO_2_ atmosphere. The oxysterols mixture, 20 μM in ethanol, was then added in the medium and cells incubated for 3 h. Well contents were collected and centrifuged at 400 × g for 5 min at 4°C. Supernatants were discarded and cells washed/centrifuged twice with cold PBS. Finally, the pellets were suspended with 150 μL of complete lysis buffer (Cell Lytic M, Sigma) with protease and phosphatase inhibitors (COmplete Ultra tablets and Mini, PhosphoSTOP, Roche) and incubated on ice for 30 min (by vortexing every 10 min). At the end, lysates were centrifuged at 15 000 × *g* for 5 min at 4°C to remove any particles or cell debris and the supernatants were collected for protein analysis. The protein concentration was determined by the Bradford protein assay,^39^ and 20 μg of protein sample were subjected to SDS‐page and western immunoblotting as previously described.^40^ The antibodies used were anti‐pp38 (1:1000 dilution), anti‐p38 (1/1000 dilution), anti‐pJNK 1/2 (1:200 dilution), anti‐JNK 1/2 (1:200 dilution), and goat anti‐rabbit IgG conjugated to horseradish peroxidase (1:2000 dilution). The blots were exposed to Hyperfilm‐ECL and developed with the ChemiDoc XRS Imager (BioRad). Protein bands were quantified using Image J software.

### 2.7 Statistical Analysis

The statistical significance of results was evaluated by one‐way ANOVA followed by Bonferroni's multiple comparison posttest using GraphPad Prism V5 (GraphPad Software, San Diego, CA, USA). *p* Values of <0.05 were considered statistically significant.

## 3 Results

### 3.1 Effect of Olive Oil Phenolics on Oxysterol‐Induced Cytokine Secretion

**Figure 1:**
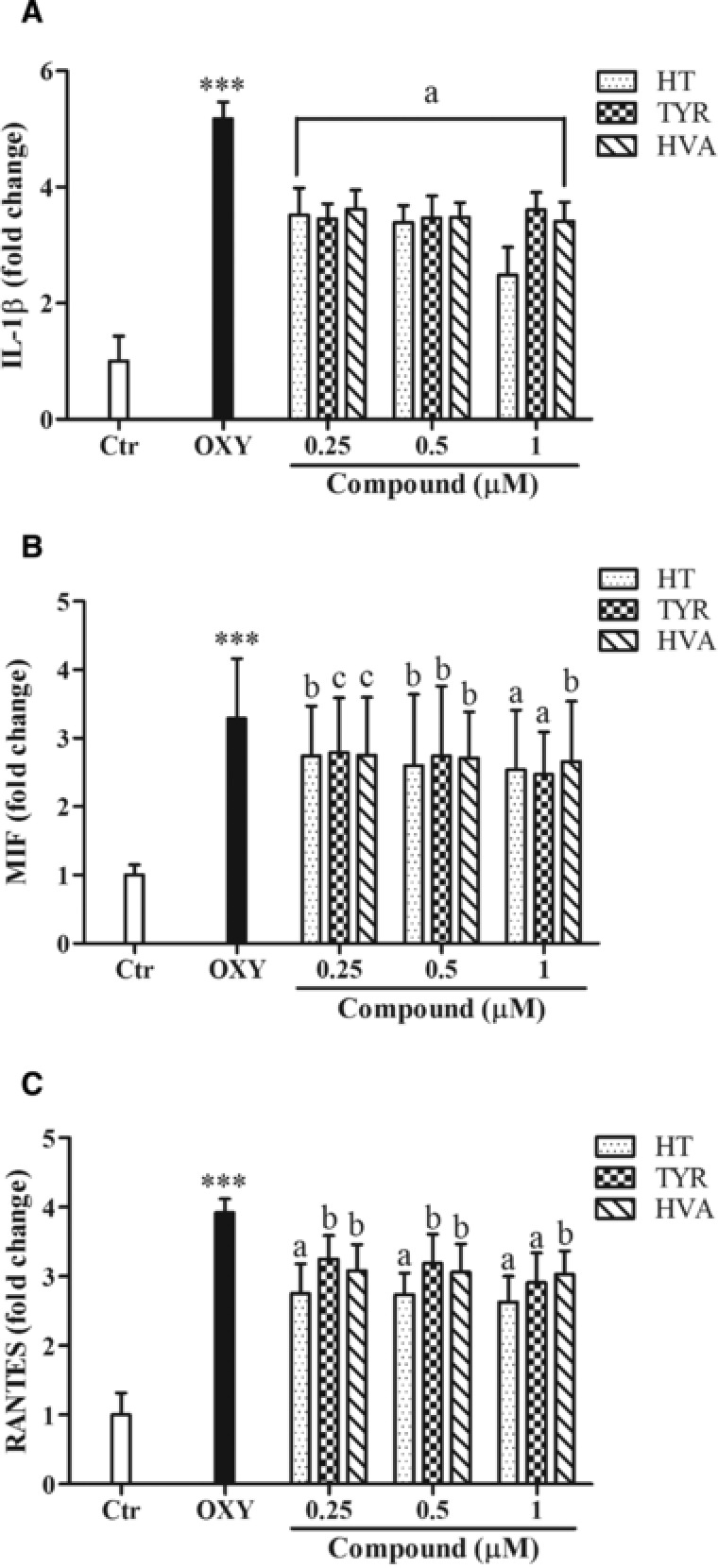
Effects of HT, TYR and HVA (0.25–0.5–1 μM) on the proinflammatory cytokines IL‐1β (A), MIF (B), and RANTES (C) secretion in PBMCs treated with the oxysterols mixture 20 μM for 24 h. Each column represents the mean ± SD of six independent experiments. ^***^ = *p* < 0.001 versus Ctrl; a = *p* < 0.001, b = *p* < 0.01, c = *p* < 0.05 versus oxy. Control value: IL‐1β = 91 pg/mL, MIF = 770 pg/mL, RANTES = 2800 pg/mL.

After an initial screening with 36 cytokines with a human cytokine proteome profiler array, three cytokines that were most significantly modulated by oxysterols were selected for further study. The levels of 3 selected cytokines (IL‐1β, MIF, and RANTES) were further subjected to quantitative analysis, which indicated that a 24 h exposure of PBMCs to oxysterols (20 μM) resulted in a significant increase in the secretion of the proinflammatory cytokines/chemokines, IL‐1β (**Figure** 1A), MIF (Figure 1B) and RANTES (Figure 1C) in PBMCs. Pretreatment with HT, TYR, and HVA (0.25, 0.5, 1 μM), significantly reduced the secretion of proinflammatory cytokines/chemokines induced by the oxysterols.

### 3.2 Olive Oil Phenolics Inhibit Oxysterol‐Induced Intracellular ROS Production

**Figure 2:**
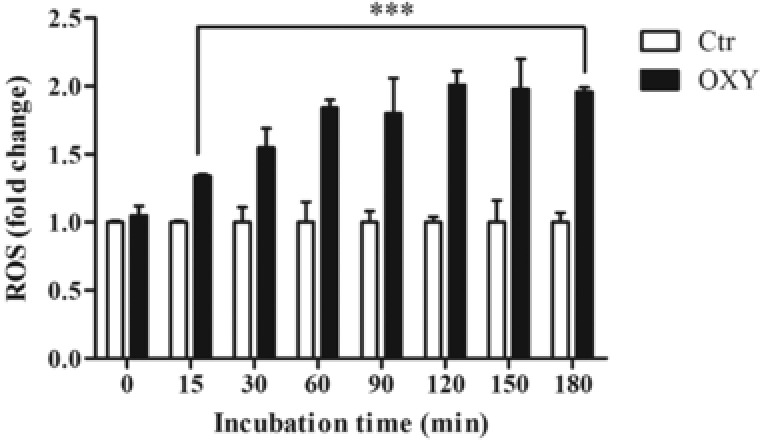
Intracellular ROS production (expressed as fold increase) in PBMCs treated with the oxysterols mixture 20 μM for different incubation times (15–180 min) using the fluorescence probe DFC‐DA 10 μM for 30 min. Each column represents the mean ± SD of six independent experiments. ^***^ = *p* < 0.001 versus ctrl.

**Figure 3:**
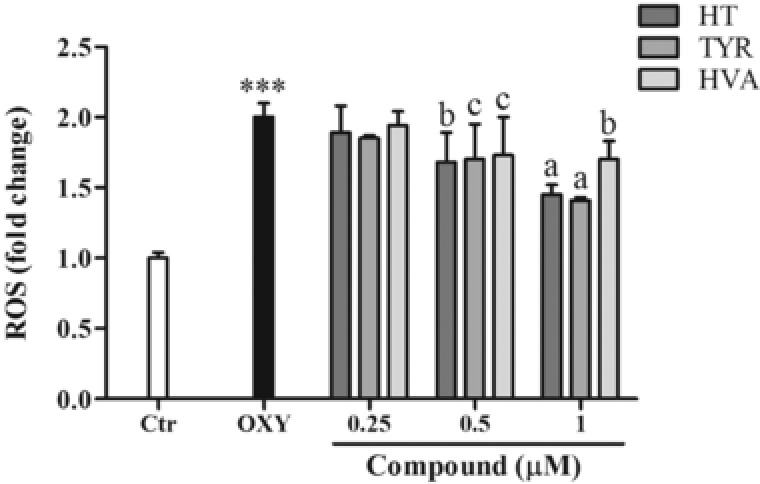
Effect of HT, TYR, and HVA (0.25–0.5–1 μM) on intracellular ROS production in PBMCs treated with the oxysterols mixture 20 μM for 2 h. Each column represents the mean ± SD of six independent experiments. ^***^ = *p* < 0.001 versus Ctrl; a = *p* < 0.001, b = *p* < 0.01, c = *p* < 0.05 versus Oxy.

Oxysterols (20 μM) also significantly increased intracellular ROS production over a 180 min timeframe (**Figure** 2). Pretreatment of PBMCs with HT, TYR, and HVA (0.25, 0.5, 1 μM) for 30 min prior to the addition of oxysterols led to a reduced intracellular ROS production at concentrations of 0.5 and 1.0 μM (**Figure** 3), with HT and TYR significantly more active than HVA.

### 3.3 Effect of Olive Oil Phenolics on Redox‐Sensitive Pathways Involved in Oxysterol‐Induced Cytokine Production

**Figure 4:**
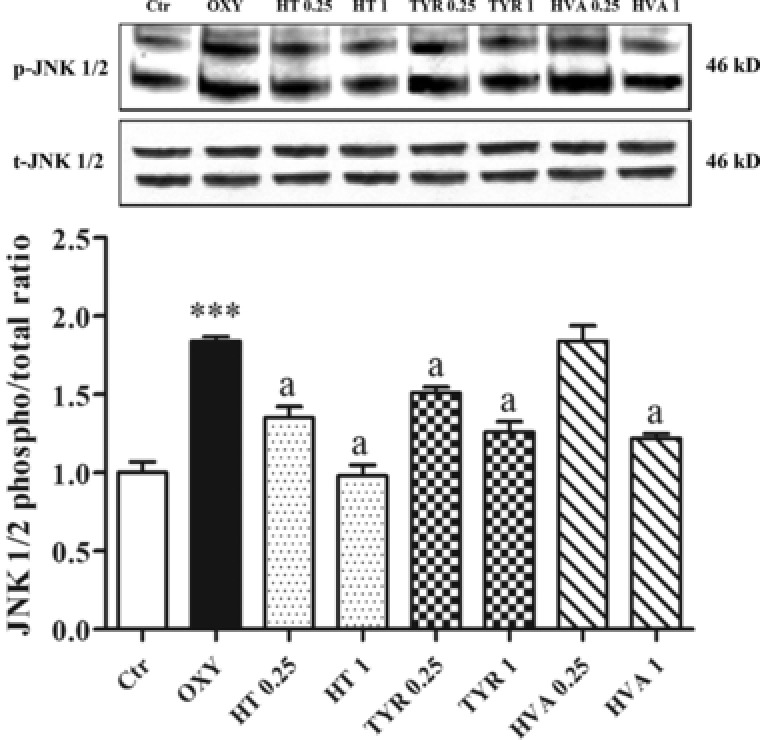
Modulation of JNK 1/2 in PBMCs pretreated or not for 30 min with HT, TYR, and HVA (0.25–1 μM) and incubated with the oxysterols mixture 20 μM for 3 h. Each column represents the mean ± SD of three independent experiments. ^***^ = *p* < 0.001 versus Ctrl; a = *p* < 0.001 versus Oxy.

**Figure 5:**
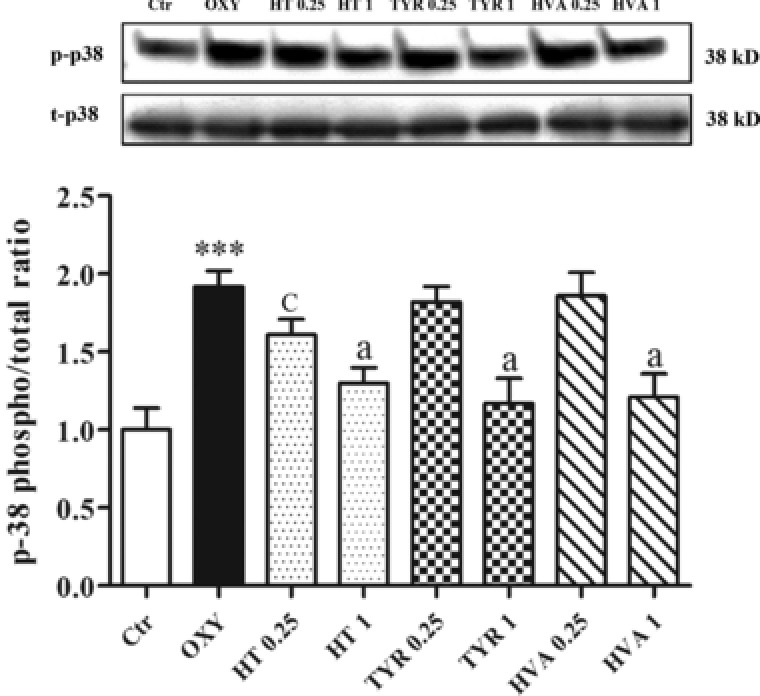
Modulation of p38 in PBMCs pretreated or not for 30 min with HT, TYR, and HVA (0.25–1 μM) and incubated with the oxysterols mixture 20 μM for 3 h. Each column represents the mean ± SD of three independent experiments. ^***^ = *p* < 0.001 versus Ctrl; a = *p* < 0.001, c = *p* < 0.05 versus Oxy.

The oxysterol mixture induced an increase in the level of the phosphorylated forms of JNK 1/2 (p‐JNK 1/2) in PBMCs following 3 h of exposure to PBMCs. All polyphenols were shown to significantly attenuate these increases with the exception of HVA, at 0.5 μM (**Figure** 4). Similarly, oxysterols also induced the phosphorylation of p38 in PBMCs, with all phenolic compounds counteracting these effects at the highest concentration and HT also active at 0.25 μM (**Figure** 5).

## 4 Discussion

Cholesterol oxidation products, termed oxysterols, may either originate endogenously, through enzymatic or nonenzymatic reactions, or may derive from the diet. As regards exogenous sources of oxysterols, foods containing cholesterol are susceptible to oxidation: oxidative reactions occur during food processing, mainly on exposure to heat treatment and during long‐term storage.^41^

They are involved in physiological processes such as the regulation of cholesterol homeostasis, but it is well established that oxysterols have mostly detrimental biological activities. They provoke an imbalance of the ratio between oxidative and reductive biochemical reactions (oxidative stress) that acts on all organism levels, from cell signaling to disease expression through upregulation of inflammation, apoptosis, and fibrosis.^7^, ^42^

Pathological accumulation of oxysterols may contribute in fact to the onset and especially to the development of major chronic diseases in which inflammation, but also oxidative damage and to a certain extent cell death, are hallmarks and primary mechanisms of progression. Indeed, certain oxysterols exercise strong prooxidant and proinflammatory effects at concentrations detectable in the lesions typical of atherosclerosis, neurodegenerative diseases, age‐related macular degeneration, and other pathological conditions characterized by altered cholesterol uptake and/or metabolism.^6^

Several studies in animals and humans have shown that dietary oxysterols, after digestion can be absorbed from the gut and transported into the circulation within chylomicrons and other lipoproteins.^43^, ^44^ Furthermore, the presence of oxysterols in plasma can derive from the oxidation of endogenous cholesterol through enzymatic or spontaneous reactions.^7^

Oxysterols have been found at increased levels in the plasma of hypercholesterolemic subjects and have been linked with the atherosclerotic process.^45^

This study focused on the protective effect of pure olive oil phenolics, which can be found in the blood stream after absorption (HT, TYR, and HVA) against the prooxidant and proinflammatory activity of oxysterols in immune blood cells.

The phenolic compounds used in our study are bioavailable,^46^, ^47^, ^48^, ^49^ as shown in numerous studies, and the concentrations used in this study are physiologically relevant (0.25–0.5–1 μM).^50^

PBMCs separated from whole blood of healthy volunteers were used for this study; these are composed by lymphocytes, monocytes and macrophages, critical components in the immune system to fight inflammation and source of proinflammatory molecules.

PBMCs cells were treated with an oxysterols mixture composed by the most widely represented oxysterols in plasma of hypercholesterolemic subjects: 7α‐HC, 7β‐HC, 7‐KC, 5α,6α‐EC, 5β,6β‐EC, triol, and 25‐HC^38^ at pathologically relevant concentration (20 μM). In fact, oxysterols in human plasma or serum may vary from about 1 μM (0.05% of total cholesterol) in healthy subjects to 20–30 μM (0.5‐0.75% of total cholesterol) in diseased individuals, but much higher concentrations of plasma oxysterols have also been reported.^4^, ^45^, ^51^

The first intent was to examine the ability of PBMCs to produce cytokines and chemokines in the presence of oxysterols, in particular, IL‐1β, MIF, and RANTES, usually involved in proinflammatory processes; the oxysterols mixture was able to significantly increase the secretion of all cytokines/chemokines analyzed. Since it has been suggested that oxysterols may increase the levels of cytokines by modulating redox‐sensitive pathways,^52^ the oxidative status of PBMCs treated with the oxysterols mixture was then measured. An early increase (after 15 min) of intracellular ROS production was observed in PBMCs challenged with oxysterols compared to control. At moderate concentrations, ROS may act as second messengers in signal transduction, by modulating redox‐sensitive MAPK; these kinases have been already reported to be activated by various stress stimuli, including treatments with different oxysterols, 7‐KC and 25‐HC^52^, ^53^ and they have been also implicated in oxysterol‐induced cytokine secretion and apoptosis.^6^, ^52^, ^54^

In the experimental conditions of this research, the oxysterols mixture induced a significant increase of both JNK and p38 phosphorylation suggesting their involvement in cytokine secretion.

These results are in accordance with other experimental studies; for example, in human monocytic cells, 7β‐HC and 25‐HC, but also 7‐KC to a lesser extent, are potent in vitro inducers of IL‐1β, IL‐8, TNF‐α, and MIP‐1β, as well as of other inflammatory molecules.^55^ The same study demonstrated that IL‐8 secretion was associated with activation of the ERK 1/2 signaling pathway.^55^ The oxysterols 7‐KC and 25‐HC have also been observed to enhance IL‐1β, IL‐6, IL‐8, and TNF‐α mRNA and secretion levels, in a dose‐dependent manner, although to different extents. These effects were associated with increased ROS production, and a net phosphorylation of MAPK (ERK 1/2, JNK, p38) and NF‐κB activation also occurred.^52^

Upregulation of IL‐1β is another important event, because this cytokine increases the surface expression of endothelial adhesion molecules, by facilitating inflammatory cells attachment to the artery endothelium. Expression and synthesis of IL‐1β were found to be stimulated by 25‐HC in human macrophages, through the involvement of the liver X receptor, as well as, but less strongly, by 27‐hydroxycholesterol.^56^ IL‐1β secretion was also markedly induced by 7β‐HC, 7‐KC, and 7α‐HC in human promonocytic cells U937 and U4^57^, ^58^ and in human umbilical vein endothelial cells (HUVECs).^59^ Production of the proinflammatory cytokines TNF‐α and IL‐1β is also induced by 25‐HC in adherent human peripheral blood mononuclear leukocytes, through phosphorylation of p38 MAPK.^60^ In the literature there is not any specific correlation between oxysterols and RANTES and MIF secretion; but the increased RANTES and MIF expressions have been associated with a wide range of inflammatory disorders and pathologies, such as inflammatory bowel disease and atherosclerosis where oxysterols exert a pivotal role. RANTES and MIF are cytokines with chemokine‐like function and critical mediators of the host immune and inflammatory response; they are thought to act by recruiting and promoting leukocytes infiltration to sites of inflammation.^61^, ^62^

In this study, it was demonstrated that all phenolic compounds HT, TYR, and HVA at all tested concentrations, were able to inhibit oxysterol‐induced proinflammatory cytokines production in ex vivo immune cells. According to the hypotesis that proinflammatory cytokines release may be induced by changes in intracellular redox status, it was observed that, in human PBMCs treated with the oxysterols mixture, simple phenols were able to inhibit ROS production significantly from the concentration of 0.5 μM as well as to suppress redox‐based MAPK phosphorylation (JNK, p38).

In the literature there are several studies that confirmed the anti‐inflammatory activity of olive oil phenolics in different districts such as in blood cells. Differential anti‐inflammatory effects of phenolic compounds from extra virgin olive oil were identified in human whole blood cultures: OL‐glycoside and CA decreased the concentration of IL‐1β and kaempferol decreased the concentration of PGE2 induced by LPS.^15^

A recent study reported similar results in macrophages where oil phenolic extract exerted their protective effects against LPS‐induced oxidative stress and inflammatory responses.^24^, ^25^ The olive oil phenolic extract significantly decreased nitric oxide (NO) and ROS production and in addition significant downregulated iNOS, COX‐2, reduced MAPK (JNK, p38) phosphorylation and prevented the nuclear NF‐κB traslocation.

HT inhibits iNOS and COX‐2 expression in LPS‐stimulated J774 cells at the transcriptional level by preventing the activation of NF‐κB, signal transducer and activator of transcription 1 (STAT‐1α) and interferon regulatory factor 1 (IRF‐1)^16^ and TYR prevented RAW 264.7 macrophages activation induced by gliadin and INFγ.^63^

A recent study conducted by Palozza et al.^52^ investigated the ability of lycopene, a strong antioxidant compound present in tomato, to prevent oxysterols induced proinflammatory cytokine cascade in human macrophages. Lycopene prevented oxysterol‐induced increase in proinflammatory cytokines (IL‐1β, IL‐6, IL‐8, TNF‐α) secretion and expression, such an effect was accompanied by an inhibition of oxysterols‐induced ROS production and MAPK activation (ERK 1/2, JNK, and p38). In our study, olive oil phenolics showed similar capabilities against the harmful effects of the oxysterols mixture in ex vivo blood cells.

Further studies are needed to clarify the mechanism by which the phenolic compounds exert their protective action; however, data obtained in these experimental systems, suggested that these compounds act primarily by counteracting the initial stages of the prooxidant and proinflammatory effects of oxysterols, inhibiting the formation of ROS and then all subsequent cascading effects.

**Abbreviations**
25‐HC: 25‐hydroxycholesterol
5α,6α‐EC: cholesterol 5α,6α‐epoxide
5β,6β‐EC: cholesterol 5β,6β‐epoxide
7‐KC: 7‐ketocholesterol
7α‐HC: 7α‐hydroxycholesterol
7β‐HC: 7β‐hydroxycholesterol
COX‐2: cyclooxygenase‐2
DCFH‐DA: 2′,7′‐dichlorofluorescein diacetate
EVOO: extra virgin olive oil
HT: hydroxytyrosol
HVA: homovanillic alcohol
ICAM‐1: intracellular adhesion molecule‐1
iNOS: inducible nitric oxide synthase
MAPK: mitogen activated protein kinase
MIF: macrophage migration inhibitory factor
MIP‐1β: macrophage inflammatory protein‐1β
NF‐κB: nuclear factor kappa‐light‐chain‐enhancer of activated B cells
NO: nitric oxide
PBMC: peripheral blood mononuclear cell
RANTES: regulated on activation, normal T cell expressed and secreted
ROS: reactive oxygen species
TNF‐α: tumor necrosis factor‐α
triol: cholestane‐3β,5α,6β‐triol
TYR: tyrosol
VCAM‐1: vascular cell adhesion molecule‐1

## Conflict of Interest

The authors have declared no conflicts of interest.
